# The challenges and opportunities of translating best practice immunisation strategies among low performing general practices to reduce equity gaps in childhood immunisation coverage in New Zealand

**DOI:** 10.1186/s12912-017-0226-2

**Published:** 2017-06-12

**Authors:** Nikki M. Turner, Nadia A. Charania, Angela Chong, Joanna Stewart, Lynn Taylor

**Affiliations:** 1 Department of General Practice and Primary Health Care, University of Auckland, 261 Morrin Road, St. Johns, Auckland, 1072 New Zealand; 2 Department of Public Health, Auckland University of Technology, 640 Great South Road, Manukau, Auckland, 2025 New Zealand; 3 Department of Biostatistics and Epidemiology, University of Auckland, 261 Morrin Road, St. Johns, Auckland, 1072 New Zealand

**Keywords:** Immunisation, Nursing, Paediatrics, Practice management, Primary care, Quality of care

## Abstract

**Background:**

Immunisation coverage rates vary considerably at the local level across New Zealand and challenges remain with effectively translating best available research evidence into public health practice. This study aimed to translate best practices from high performing general practices into strategies to improve childhood immunisation coverage among low performing practices.

**Methods:**

An intervention study was undertaken of general practices with low immunisation coverage rates and a high percentage of the enrolled population being of Māori ethnicity. Intervention groups received customised action plans and support for a 12 month period while control groups received ‘business as usual’ support. Structured interviews were conducted with key informants from all participating practices to understand current aspects related to childhood immunisation delivery and surveys were conducted to understand how the intervention worked. Collected data were thematically analysed.

**Results:**

Ten sites were randomised to either intervention (*n* = 6) or control group (*n* = 4). Positive aspects of childhood immunisation delivery included high prioritisation at the practice and staff being pro-immunisation and knowledgeable. Key challenges experienced included inaccurate family contact information and discrepancies with referral processes to other providers. Other challenges noted were building rapport with families and vaccine hesitancy. The action plans included various strategies aimed to improve processes at the practice, contact and engagement with parents, and partnership development with local service providers.

**Conclusions:**

Creating customised action plans and providing support to providers were considered as helpful approaches when attempting to improve childhood immunisation coverage rates. Our study supports the notion that one strategy will not solely by itself improve childhood immunisation rates and highlights the importance of having a toolkit of strategies from which to draw from.

## Background

Childhood immunisation against vaccine-preventable diseases is often cited as one of the most successful preventive health interventions and a highly cost-effective healthcare activity [1]. In New Zealand, the National Immunisation Schedule (NIS) consists of publicly funded vaccination for all children at certain milestone ages [2]. Inequities in immunisation uptake by deprivation and ethnicity have been reported across New Zealand, particularly for some ethnic groups and children from backgrounds of higher deprivation [3–5]. The historically suboptimal childhood immunisation coverage rates and delayed immunisation timing has sparked the implementation of numerous strategies to improve uptake rates and reduce equity gaps [6]. The national childhood immunisation program is largely implemented in General Practitioner medical centres by vaccinating nurses during well-child visits, with additional immunisations provided by outreach services [6]. Despite overall immunisation coverage rates improving nationally and disparity gaps closing, these rates vary considerably among general practices at the local level [5–7].

Literature suggests many factors at the practice level that may contribute to this variation, including the priority placed on immunisation and staff confidence and knowledge related to immunisation [6, 7]. Structural and organisational aspects of general practices, such as the type of practice management system used and not experiencing any staff shortages, have also been attributed to impacting immunisation coverage and timeliness at the local level [5, 7]. Barriers to achieving high immunisation coverage have also been associated with parental characteristics and conditions within which families live [8]. Parental apathy, fear, ambivalence and experiencing difficulties accessing immunisations have been perceived as barriers to immunisation by healthcare providers [7]. Studies have suggested various strategies and practical examples to overcome the identified challenges associated with improving and sustaining immunisation coverage rates. For instance, developing programme infrastructure and collecting accurate immunisation data have been suggested [9]. Having strong leadership in place, focusing on parental education and communication, and developing partnerships with other service providers are examples of some other initiatives [9, 10].

Earlier New Zealand research identified characteristics associated with general practices that obtained and maintained high childhood immunisation coverage rates and timeliness of delivery [11]. Effectively translating the best available research evidence into public health practice remains suboptimal given the complex nature of this process [12, 13]. Thus, the purpose of this study was to present the experiences of translating the knowledge gained from these high performing general practices into strategies to improve childhood immunisation coverage among low performing practices. The overall aim of this study is to further improve childhood immunisation coverage and reduce immunisation inequities.

## Methods

### Study design, sample population & recruitment process

**Fig. 1:**
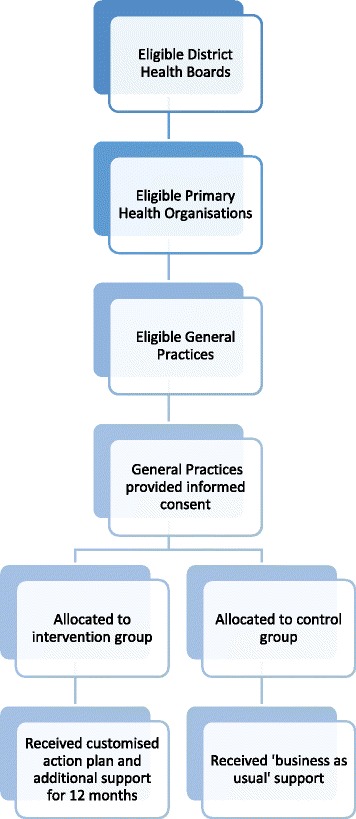
Flowchart of intervention study design and recruitment process

This study presents the qualitative component of an intervention study that was undertaken of general practices from regions across New Zealand with low childhood immunisation coverage rates (unpublished, Immunisation Advisory Centre 2012) (Fig. 1). To inform the recruitment process, immunisation coverage reports were used that are based on the National Immunisation Register (NIR) data and published by the Ministry of Health. The NIR is a database that automatically records each child born and when immunisations are given (entered by the vaccinator directly into the practice management system). In accordance with the NIR, immunisation status was defined as “fully immunised for age” if the child had received all of the age-appropriate vaccines by the time the milestone age was reached [2].

The organisations approached and invited to participate were those within District Health Board (DHB) regions that reported total childhood immunisation coverage rates lower than national average, according to NIR reports between September 2012 and March 2013 (i.e. 85.4%), and had a potential pool of more than 500 eligible children. The DHB itself or a Primary Health Organisation (PHO) operating within the DHB region acted as local health delivery partner organisations for this study. If initial contact was made with a DHB, the DHB subsequently put the researchers in contact with appropriate PHOs within the respective region.

General practices were ranked in order of immunisation coverage percentage within their local region. The practices in the lowest two socioeconomic quintiles for their local region with an enrolled population higher than the national average for Māori ethnicity (i.e. greater than 15%) were eligible to participate. General practices with a reported decline rate higher than twice the national average (i.e. greater than 10%) or had with small numbers of children (less than 20) born in the last 12 months were not eligible to participate. Consenting practices were subsequently randomised to either the intervention or control group using a randomly generated order, blocked by DHB region. Ethical approval to conduct this research was granted by the Health and Disability Ethics Committees of the Ministry of Health (reference: 12/NTA/90).

### Data collection

Structured interviews were conducted with key informants from all participating practices to gain an understanding of the current situation regarding childhood immunisation delivery. Selected staff members (herein referred to as practice champions) were those whose primary responsibility was immunisation delivery and were either a practice nurse, practice manager, or clinical leader/nurse manager. Interview questions were based on areas of practice that are most relevant to childhood immunisation delivery outlined in academic literature. Questions were asked about the local environment, the practice team, staff roles and responsibilities, and practice systems and process for childhood immunisations. A combination of closed- and open-ended questions were included and prompts were used to elicit elaboration from participants. The interviews were conducted in person by one of the researchers (LT or AC) during the period from May to November 2013 and ranged from 1 h and a half to 2 h in duration. With the participants’ permission, responses were transcribed verbatim during the interviews.

General practices allocated to the control group received ‘business as usual’ support from their respective PHO and DHB. Those sites allocated to the intervention group each received a customised action plan with strategies to improve childhood immunisation delivery. A template was used to create the action plans that included goal statements, specific activities to be implemented, and indicators of progress for every strategy suggested. These action plans were created by the practice champion and one of the researchers (AC), along with input from the local PHO and/or DHB coordinators where appropriate. The customised action plans comprised of 11 to 21 strategies per practice and were based on areas for improvement gleaned from the baseline structured interviews and identified immunisation best practices [11] (Table 1).

**Table 1:** Childhood immunisation best practices identified from high performing general practices in New Zealand [adapted from 11]

Category	Best practice theme	Description
Engagement with parents	Connection and communication with parents/caregivers	• Contact families during the antenatal period or early post-natal period (either directly or indirectly through antenatal providers) • Use creative ways to build and maintain rapport with families (e.g. friendly welcome letters, health promotion days, etc.) • Improve access to the general practice for immunisation visits (e.g. extended evening hours, weekend hours, etc.)
Practice-based processes	Practice internal systems and processes	• Develop a systematic approach for scheduling immunisation visits (e.g. pre-booking, reminders, recalls) • Use electronic practice management system to facilitate opportunistic vaccinations
Practice-based processes and partnership development with local service providers	Connection with difficult to find children	• Reduce missed opportunities (e.g. identify children overdue for immunisations, check family contact information, etc.) • Offer ‘in-kind’ support to encourage attendance (e.g. transportation and supermarket vouchers) • Contact other local organisations and providers to locate families • Referral to outreach immunisation services
Practice-based processes	Use of electronic tools	• Effective use and knowledge of electronic practice management system and interface with the National Immunisation Register (NIR) • Cautious use of ‘decliner’ or ‘non-responder’ immunisation codes • Use additional electronic support tools (e.g. DrINFO audit tool, Patient Dashboard, etc.)
Practice-based processes	Practice immunisation targets and performance monitoring	• Monitor practice immunisation coverage rates against internal and national targets
Practice-based processes	Staff training and continuing education	• Commitment to attending staff education and training opportunities

Additional support (via emails, phone calls, and in-person visits) to implement the suggested strategies was provided by a researcher (AC) for a 12 month intervention period. Upon completion of the intervention phase, practice champions were asked to complete a brief survey to provide feedback. Questions were asked about how the intervention worked (e.g. easiest and hardest strategies to implement, barriers to implementation, etc.) and the level of support provided.

### Data management and analyses

The collected data were manually transcribed into electronic format and imported into QSR NVivo® version 10 (QSR International Pty Ltd., Doncaster, Victoria, Australia) for management and analyses. Thematic analysis was undertaken to identify key themes from the structured baseline interviews and feedback surveys. Data were deductively analysed using a template organising approach in which the questions were used as a coding template [14, 15]. A thematic analysis using inductive coding was performed on the text of the action plan documents to systematically identify emerging patterns [15]. Data analysis was an iterative process completed multiple times and memos were written throughout the coding process [15].

## Results

The target was to recruit 40 general practices in total, with 20 practices each being randomised to the intervention and control group [11]. Five DHBs were initially selected and subsequently nominated 12 PHOs, of which 9 completed the eligibility template to identify potential general practices. In total, 32 sites were deemed eligible to participate and were approached by the respective health delivery partner. Recruitment was a challenge as 22 eligible sites opted to decline. The remaining ten sites (coded A-J for anonymity) were successfully recruited and randomised to either the intervention (*n* = 6) or control group (*n* = 4) (Table 2). In the period leading up to the recruitment phase, many regions had already committed a significant amount of effort to improving immunisation coverage rates and thus were unwilling to participate. Moreover, concerns related to staff shortages and increased workload were commonly reported. Due to low recruitment numbers, the study did not progress as a randomised controlled trial as originally planned and instead focused on the qualitative component of an intervention study.

**Table 2:** Site characteristics of participating general practices (*n* = 10)

Study Code	Group Allocated to	District Health Board region	Collected at Eligibility Determination			Collected during Baseline Interviews		
			Immunisation coverage rate (%)^a^	Enrolled population of Māori ethnicity (%)	Enrolled population declined immunisation (%)	Number of doctors	Number of nurses	Number of registered patients
A	Intervention	Bay of Plenty	83	52	0	1	2	1979
B	Intervention	Northland	80	63	9	6	4	8116
C	Intervention	Lakes	73	41	0	4	3	3500
D	Intervention	Northland	67	92	8.3	9	13	6340
E	Intervention	Lakes	67	65	0	2	4	3861
F	Intervention	Northland	77	25	9	13	12	11,700
G	Control	Bay of Plenty	77	32	4.5	5	3	5500
H	Control	Northland	82	44	0	7	5	6100
I	Control	Lakes	63	63	6.3	5	5	5842
J	Control	Northland	60	95	0	2	3	1333

^a^National Immunisation Register (NIR) reported immunisation coverage at the milestone age of 8 months of age (3 month reporting period)

### Enablers and barriers to childhood immunisation delivery

With regards to the practice team and priorities, most participants reported that childhood immunisation was of high importance and regularly discussed at team meetings. Additionally, high confidence and knowledge levels related to immunisation were reported, with many staff members actively partaking in training opportunities and keeping required certifications current. Participants also reported that staff views were generally pro-immunisation as most staff members themselves received all recommended vaccinations.

“*All pro-immunisations, all staff pro flu vaccine … think all staff children [are] vaccinated*” (Participant #1).

The vast majority of respondents discussed challenges associated with the low socioeconomic status of the practice patient population and maintaining accurate contact information of patients due to the transient nature of some families. Participants frequently expressed difficulties in locating children due for immunisations because families often relocated and did not amend their contact information. Moreover, many participants reported transportation barriers that impacted the ability of families to attend immunisation appointments.

“*[Community] in general is a low socio-economic area. Biggest challenges would be transient families, no working phones or incorrect information*” (Participant #9).

“ … *families with one car, waiting for partner to come home before can bring young child in … whānau (extended family) living under one roof as extended family and only have one car*” (Participant #1).

Some participants reported that practice staff themselves would undertake home visits if possible to immunise children. Referrals to outreach immunisation services and other well-child health providers were routinely made in situations where practice staff could not locate children overdue for scheduled immunisations. However, participants reported discrepancies with this referral process and issues with the coverage of the outreach services.

Several reported challenges related to parental vaccine refusal and hesitancy, including anti-immunisation beliefs, competing priorities of parents, and lack of vaccine-related education and health literacy of parents.

“*Lack of education of why we immunise, lack of education of severity of disease that vaccine can prevent. Not a priority, scared child will cry or get sick and they [parents] are left with crying baby*” (Participant #8).

Another common impediment related to building rapport and relationships with family members as participants reported that efforts aimed at engaging families were generally limited to national immunisation awareness week activities. Other common challenges related to the lack of formal engagement with other service providers and issues with internal practice data management systems and processes.

### Action plans to improve childhood immunisation coverage

Strategies to improve childhood immunisation coverage were organised into categories depending on whether the strategy aimed to improve processes at the practice, engagement with parents, or partnership development with local service providers.

**Practice-based processes** The most frequently implemented strategies were related to maintaining accurate contact details of patients as this was reported to be a key challenge experienced. Efforts were made by the reception and clinical staff to consistently confirm the contact details of patients visiting the practice. Also, complimentary ‘change of address’ cards were offered to encourage parents to update their mailing address if they relocated. Moreover, using the social networking site, Facebook, as a tool to engage with and contact highly mobile families was suggested to all of the practices.

Strategies were instigated to improve the efficiency of the systems used to collect and manage practice data. For instance, the process to enrol newborns at the practice and capture reasons for declining immunisations was clarified. Moreover, any glitches involving the interface between the internal practice management system and the national data management system (i.e. NIR) were addressed, such as receiving duplicated messages and sending notifications when a child’s status changed.

Strategies were suggested to maintain or increase the prioritisation of childhood immunisations in the team. For instance, more frequent practice team meetings were scheduled during which immunisation was a tabled agenda item. Also, newly available video resources about immunisations were distributed to practices to engage clinical staff.

**Engagement with parents** Initiatives directed at communication, relationship building, and education with parents were common. When notified of a new birth, friendly phone calls were made to parents to congratulate them and welcome them to the general practice. Some practices also mailed a welcome letter to parents of newborns, along with pamphlets about immunisation and practice enrolment forms. To help make the immunisation event a positive experience, clinical staff contacted relevant organisations to obtain resources and samples of baby products that were assembled into packages to giveaway to parents. Clinical staff also made efforts to call parents to provide reassurance and answer any questions after their child’s first immunisation visit. Numerous efforts to remind parents of when their child’s immunisations were due (reminders) or late (recalls) were implemented using a combination of phone calls, text messages, and letters. One practice also created refrigerator magnets that parents could personalise to include the dates of their child’s immunisation appointments.

Strategies to improve immunisation opportunities by better accommodating parents’ demanding schedules were implemented, such as offering weekend and flexible drop-in immunisation clinics. Improving access to immunisation related information was seen as an important tool to improve the education and health literacy of parents. Resources, such as videos, displays, and pamphlets, were set up in waiting rooms and tailored to address parents’ questions (e.g. the costs of non-funded immunisations) and alleviate voiced concerns (e.g. safety of multiple injections given at the same visit).

**Partnership development with local service providers** The importance of communication with other local service providers was noted with strategies directed towards developing partnerships with midwives, community well-child providers, and allied healthcare workers. Clinical staff proactively approached local service providers to arrange meetings, formalise relationships and improve communication to keep immunisation messages in the forefront, reinforce a team approach to childhood immunisations and improve referral processes.

**Experiences with implementing action plans** Support (via emails, phone calls, and in-person visits) to implement the action plan was provided for a 12 month period. Upon completion of the intervention period, due to staff turnaround and availability, four practice champions completed the surveys to provide feedback. Some participants voiced the value of having support to review their practice’s immunisation processes and create an action plan as innovative strategies were proposed. Contact method and frequency was tailored to each practice as the study progressed and participants reported that the type, level, and frequency of support provided was suitable.

“*This process has been good. Being independent with no agenda, good as looked with fresh eyes [and] came up with some good ideas … ”* (Participant #5).

“*Email [is preferred] as it gives us time to go over things first … time is one of our biggest factors, so phone and face-to-face are time consuming for us*” (Participant #4).

Participants reported that some of the strategies were particularly feasible to implement and were readily adopted into routine practice, such as consistently confirming contact details of patients, obtaining baby samples for giveaways, and creating resources to address parental vaccine-related beliefs. Participants also conveyed the positive feedback they received about the friendly phone calls that were made to welcome parents or provide reassurance.

Conversely, it was unclear how well supported and integrated other strategies were, as some were either discontinued or deemed to not be a priority. Most notably, using Facebook as a tool to connect with difficult to find families was not adopted in any of the participating practices, despite initial interest. However, one practice used Facebook as an avenue to advise community members about a local measles case and urge parents to have their children fully immunised which positively resulted in many calls to the practice.

The most commonly reported barriers related to changing daily practice included an already demanding staff workload, along with competing priorities, and generally being reluctant to change.

“ … *mindset of leaders working on the Action Plan [were] not users of Facebook themselves. So, needed to have the right person to implement for them and see how best to structure* … ” (Participant #6).

Thus, participants described factors that enabled the implementation of strategies, including the provision of a sound rationale for the proposed change in order to gain interest, protected staff time to focus on immunisation activities, and support, training, and information as necessary.

“*Making sure there is time, support and training, information as to why the change*” (Participant #5).

### Trends in childhood immunisation coverage

NIR immunisation coverage data at the 8 month old milestone age (12 month reporting period) were collected from participating general practices for the total practice population and those of Māori ethnicity (Table 3). Immunisation coverage rates from 1 month after conducting the baseline interviews were selected to account for the potential effects on immunisation delivery due to participating in the interview process. As a comparison, immunisation coverage rates were selected from 15 months after the baseline interviews to account for the 12 month intervention period plus the additional time taken to finalise the action plans. As there was considerable variability throughout the intervention period and after completion of the study, no obvious differences in immunisation coverage rates were noted between the control and intervention practices (Table 3).

**Table 3:** Change in immunisation coverage rates of participating general practices from 1 month to 15 months post baseline interviews

Study Code	Group Allocated to	Total immunisation coverage rate 1 month post baseline (%)^a^	Total immunisation coverage rate 15 months post baseline (%)^a^	Change in total immunisation coverage rate	Māori immunisation coverage rate 1 month post baseline (%)^a^	Māori immunisation coverage rate 15 months post baseline (%)^a^	Change in Māori immunisation coverage rate
A	Intervention	85%	88%	3%	83%	85%	2%
B	Intervention	81%	89%	8%	79%	91%	12%
C	Intervention	82%	82%	0%	80%	81%	1%
D	Intervention	73%	74%	1%	72%	79%	7%
E	Intervention	82%	91%	9%	81%	85%	4%
F	Intervention	82%	84%	2%	76%	82%	6%
G	Control	76%	89%	13%	69%	88%	19%
H	Control	88%	93%	5%	75%	93%	18%
I	Control	85%	89%	4%	83%	88%	5%
J	Control	69%	92%	23%	69%	92%	23%

^a^National Immunisation Register (NIR) reported immunisation coverage at the milestone age of 8 months of age (12 month reporting period)

## Discussion

The strategies included in the action plans directly corresponded to the challenges related to childhood immunisation delivery experienced by the participants. While each general practice had a customised action plan that included various strategies, several common themes emerged that relate to improving childhood immunisation coverage. These included the importance of adequate practice processes, creating a team approach to immunisation, and the commitment to engaging with parents.

The need for adequate practice processes was most notably reported in relation to providing immunisation services for families that were difficult to contact or hard-to-reach. These children may miss scheduled appointments and subsequently, will risk being under-immunised. Patient reminder and recall interventions are known to be effective in improving immunisation coverage [16]. Accordingly, the practice staff used a combination of traditional immunisation communication methods to recall patients (e.g. phone calls, letters); however, inaccurate parent contact information, especially of those that are highly mobile, posed a considerable barrier. Newer technologies, such as text messaging and email, have been suggested as feasible, effective, and well-accepted alternative avenues to contact parents [17, 18]. The potential use of social networking sites (e.g. Facebook, Twitter) has also been explored; however, similar to our study, none of the interviewed practices were using such tools to contact patients about immunisation appointments [19]. Similar to others, our study highlighted providers’ reluctance to change and the lack of provider buy-in with proposed initiatives, especially those involving social networking sites despite inferred parental acceptance [20].

Studies have reported the equity gaps in immunisation coverage among geographically hard-to-reach populations [21] and the important role played by outreach efforts in reducing the barriers to accessing necessary immunisation services for the underserved [10]. This study indicated that issues were experienced with coordinating referrals to outreach immunisation services. Although strategies helped to clarify the referral process between the general practices and outreach services, implementing a standardised referral process may alleviate the reported variability and confusion.

Maintaining the focus of providers on childhood immunisations and overcoming ‘immunisation fatigue’ is reported to be problematic as clinical staff face increasingly complex childhood immunisation schedules and struggle with parental vaccine hesitancy [10]. Given that providers’ recommendations strongly influence parental acceptance of immunisations, it is important that providers continuously prioritise immunisations and update their knowledge accordingly [10]. Developing partnerships and collaborating with other service providers that play a role in immunisations has also been suggested to stimulate interest and strengthen community-level support for immunisations [10]. Accordingly, strategies suggested in our study placed considerable focus on prioritising childhood immunisations and encouraging a team approach within the practice and with other local service providers.

Previous studies have noted various complex factors associated with parental vaccine refusal and hesitancy towards childhood immunisations [21, 22]. Parental concern and lack of education about immunisation has been often cited as a challenge [10]. Parent-centred information and education interventions, such as educational pamphlets and posters, are most commonly used to reduce parental vaccine refusal and hesitancy [23]. The action plans included various educational interventions aimed at improving vaccination literacy amongst parents to enable them to make well-informed decisions and hopefully improve vaccine demand. Building rapport and trust between the provider and parents is also critical in shaping parental attitudes towards vaccinations [24, 25]. Moreover, negative immunisation experiences and dissatisfaction with immunisation services have been associated with suboptimal childhood immunisation [22, 26]. As such, strategies were implemented at participating practices to develop a positive rapport with parents and improve their overall immunisation experience.

Overall, creating customised action plans, along with providing additional support to implement and review the proposed strategies, appeared to be a helpful approach when attempting to improve childhood immunisation coverage rates. The achieved sample size was too small to draw any conclusions and the immunisation coverage rates revealed considerable variability; however, most practices showed some improvement. Willingness to participate in this study may indicate that these practices regard childhood immunisations as a high priority and could be a factor in the improvement seen in the control group. Moreover, completing the baseline interviews may have increased awareness at all participating practices and may have contributed to improving aspects related to childhood immunisation delivery. Our study supports the notion that one initiative will not solely improve childhood immunisation rates and highlights the importance of having a toolkit of initiatives from which to draw from. Since many complex factors contribute to low immunisation rates, it is no surprise that a combination of interventions or multicomponent ones will be required to improve immunisation coverage [8]. The action plans also offered a variety of strategies tailored to address the local problems and challenges experienced by each general practice.

This study has many strengths as in-depth information was ascertained directly from those working at general practices in childhood immunisation delivery. Some of the strategies and reflections from implementing the action plans discussed herein may be useful to others aiming to improve immunisation coverage rates. However, the results may not be directly generalisable to other settings. Additionally, as key informants were nominated to participate by each general practice, the perspectives presented may not be inclusive of the insights of others in the team. It was also assumed that the key informants would share reliable and trustworthy information regarding their experiences.

Future research should continue to be directed towards better understanding how to effectively translate best practices associated with high childhood immunisation coverage into routine practice, including which combinations of interventions are most effective. Further investigation is also warranted into the perceptions of providers and parents particularly regarding the use of social networking sites and strategies of how to overcome providers’ reluctance to adopt newer technologies for the purpose of immunisation reminders.

## Conclusions

Our study aimed to translate best practices into strategies that can be implemented to improve childhood immunisation coverage among low performing general practices across New Zealand. Creating customised action plans comprised of multiple strategies and providing support appeared to be a helpful approach when attempting to improve immunisation rates. Our study supports the notion that one initiative will not solely improve childhood immunisation rates and reveals the importance of strategies that focus on the providers and general practice within their own context.
